# Concept of Embedded Dipoles as a Versatile Tool for Surface Engineering

**DOI:** 10.1021/acs.accounts.2c00173

**Published:** 2022-06-03

**Authors:** Egbert Zojer, Andreas Terfort, Michael Zharnikov

**Affiliations:** § Institute of Solid State Physics, NAWI Graz, Graz University of Technology, Petersgasse 16, 8010 Graz, Austria; † Institut für Anorganische und Analytische Chemie, Johann Wolfgang Goethe Universität Frankfurt, Max-von-Laue-Straße 7, D-60438 Frankfurt am Main, Germany; ‡ Angewandte Physikalische Chemie, Universität Heidelberg, Im Neuenheimer Feld 253, D-69120 Heidelberg, Germany

## Abstract

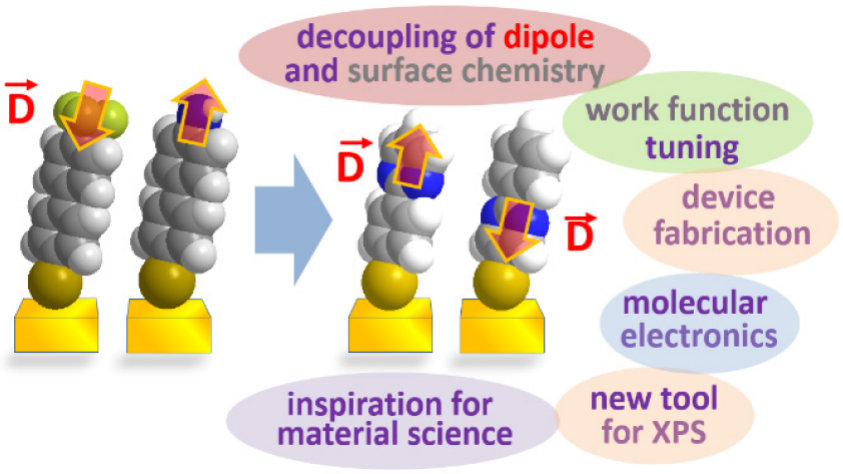

Controlling the physical and chemical properties of surfaces and interfaces is of fundamental relevance in various areas of physical chemistry and a key issue of modern nanotechnology. A highly promising strategy for achieving that control is the use of self-assembled monolayers (SAMs), which are ordered arrays of rodlike molecules bound to the substrate by a suitable anchoring group and carrying a functional tail group at the other end of the molecular backbone. Besides various other applications, SAMs are frequently used in organic electronics for the electrostatic engineering of interfaces by controlling the interfacial level alignment. This is usually achieved by introducing a dipolar tail group at the SAM–semiconductor interface. Such an approach, however, also changes the chemical character of that interface, for example, affecting the growth of subsequent layers. A strategy for avoiding this complication is to embed polar groups into the backbones of the SAM-forming molecules. This allows disentangling electronic interface engineering and the nucleation of further layers, such that both can be optimized independently. This novel concept was successfully demonstrated for both aliphatic and aromatic SAMs on different application-relevant substrates, such as gold, silver, and indium tin oxide. Embedding, for example, ester and pyrimidine groups in different orientations into the backbones of the SAM-forming molecules results in significant work-function changes. These can then be fine-tuned over a wide energy range by growing mixed monolayers consisting of molecules with oppositely oriented polar groups. In such systems, the variation of the work function is accompanied by pronounced shifts of the peaks in X-ray photoelectron spectra, which demonstrates that electrostatically triggered core-level shifts can be as important as the well-established chemical shifts. This illustrates the potential of X-ray photoelectron spectroscopy (XPS) as a tool for probing the local electrostatic energy within monolayers and, in systems like the ones studied here, makes XPS a powerful tool for studying the composition and morphology of binary SAMs. All these experimental observations can be rationalized through simulations, which show that the assemblies of embedded dipolar groups introduce a potential discontinuity within the monolayer, shifting the energy levels above and below the dipoles relative to each other. In molecular and monolayer electronics, embedded-dipole SAMs can be used to control transition voltages and current rectification. In devices based on organic and 2D semiconductors, such as MoS_2_, they can reduce contact resistances by several orders of magnitude without adversely affecting film growth even on flexible substrates. By varying the orientation of the embedded dipolar moieties, it is also possible to build p- and n-type organic transistors using the same electrode materials (Au). The extensions of the embedded-dipole concept from hybrid interfaces to systems such as metal–organic frameworks is currently underway, which further underlines the high potential of this approach.

## Key References

- Abu-HuseinT.; SchusterS.; EggerD. A.; KindM.; SantowskiT.; WiesnerA.; ChiechiR.; ZojerE.; TerfortA.; ZharnikovM.The Effects of Embedded Dipoles in Aromatic Self-Assembled Monolayers. Adv. Funct. Mater.2015, 25, 3943–3957.(1) Both the work-function engineering and modification of X-ray photoelectron (XP) spectra associated with an embedded dipole are demonstrated and rationalized.
- TaucherT. C.; HehnI.; HofmannO. T.; ZharnikovM.; ZojerE.Understanding Chemical versus Electrostatic Shifts in X-ray Photoelectron Spectra of Organic Self-Assembled Monolayers. J. Phys. Chem. C2016, 120, 3428–3437.10.1021/acs.jpcc.5b12387PMC476197326937264(2) The importance of electrostatic effects in XP spectra of thin organic films is demonstrated and rationalized.
- HehnI.; SchusterS.; WächterT.; Abu-HuseinT.; TerfortA.; ZharnikovM.; ZojerE.Employing X-ray Photoelectron Spectroscopy for Determining Layer Homogeneity in Mixed Polar Self-Assembled Monolayers. J. Phys. Chem. Lett.2016, 7, 2994–3000.2742904110.1021/acs.jpclett.6b01096PMC4976398(3) The ability of XPS to serve as a versatile tool to study the morphology of mixed SAMs is demonstrated.
- PetritzA.; KrammerM.; SauterE.; GärtnerM.; NascimbeniG.; SchrodeB.; FianA.; GoldH.; CojocaruA.; Karner-PetritzE.; ReselR.; TerfortA.; ZojerE.; ZharnikovM.; ZojerK.; StadloberB.Embedded Dipole Self-Assembled Monolayers for Contact Resistance Tuning in p- and n-Type Organic Thin Film Transistors and Flexible Electronic Circuits. Adv. Funct. Mater.2018, 28, 1804462.(4) The potential of embedded-dipole SAMs in organic electronics is demonstrated by the example of organic thin film transistors.

## Introduction

Control of the chemical and physical properties of surfaces and interfaces is one of the key challenges of modern nanotechnology. A versatile tool for meeting that challenge is the functionalization of surfaces with self-assembled monolayers (SAMs). These are ordered assemblies of rodlike molecules, which comprise an anchoring group for the attachment to the substrate, a tail group exposed to ambient or an adjacent material, and a backbone connecting the anchoring and tail groups and promoting the self-assembly.^5^ Such an architecture allows the preparation of polar SAMs, useful in the context of electrostatic engineering of interfaces in organic electronics.^6−9^ The achieved control over the energy level alignment between different functional layers can then, for example, be used to minimize charge-carrier injection barriers. A standard approach in this context is the introduction of a polar tail group, which can also comprise a fluorinated alkyl segment.^9−12^ This approach has, however, a few drawbacks. First and foremost, the introduction of a polar tail group does not only change the work function (WF) of the substrate but also redefines the chemical character and wetting properties of the surface of the SAM. In fact, it has been shown that the use of polar as well as nonpolar SAMs can affect the morphology and growth mode of a layer subsequently grown on top of the modified interface.^7,9,13−15^ Second, the growth of subsequent layers can affect the nature of the terminal tail group, changing its properties. Third, it is hardly possible to attach several subsequent polar tail groups onto the same molecule in order to maximize the achievable WF change.

An alternative to polar tail groups is the introduction of polar entities into the molecular backbones. The electrostatic properties of the resulting SAM can then be flexibly varied by adjusting the dipole moment of the embedded group, while the physicochemical character of the SAM surface remains unchanged and/or can be adjusted independently. It has also been predicted theoretically that the inclusion of multiple dipolar units into the backbone structure could trigger particularly large changes of the WF.^16^ Similarly, combining embedded dipoles with a polar tail group has the potential to generate particularly large molecular dipole moments and SAM-induced work-function changes.^17^

Apart from the obvious advantages, the embedded-dipole strategy also bears some challenges. First, the synthesis of embedded-dipole molecules is typically more demanding than attaching a polar group to an established SAM-forming molecule.^18,19^ Second, when forming embedded-dipole SAMs, one cannot directly rely on reproducing the structural parameters of the similar nonpolar systems. Therefore, particular care has to be taken to verify the quality of the formed films and to understand the details of the molecular organization within embedded-dipole monolayers. These challenges can, however, be adequately met, as will be demonstrated in the present Account. Here, we primarily focus on work performed recently in the groups of the authors. It should, however, also be mentioned that in the literature there are several other examples of embedded-dipole SAMs.^20−23^ Moreover, in the discussion we largely concentrate on thiolate SAMs on gold in view of the broad use of these systems both in basic research and in applications.^5^

## Representative Embedded-Dipole Systems

Adding functional tail groups to molecular backbones is a well-known strategy used for a variety of purposes, such as the creation of intermolecular hydrogen bonds adding to monolayer stability, the breaking of the SAM symmetry for (nonlinear) optical applications, the introduction of predefined “weak links” for lithography, etc.^5^ Apart from a study targeting the impact of the dipole position on the general properties of SAMs,^24^ to the best of our knowledge the first report specifically considering the impact of embedded dipoles on the electronic structure of SAMs was published in 2008, dealing with mid-ester substituted alkanethiolate (AT) SAMs on Au(111).^18^ The next important step was the creation of SAMs of carboranethiol isomers, where varying the position of the carbon atoms in the carboranes and the use of mixed monolayers allowed controlling the electrode WF and, thus, the performance of organic thin film transistors (OTFTs) without affecting the growth mode of subsequent layers.^20^

**Figure 1:**
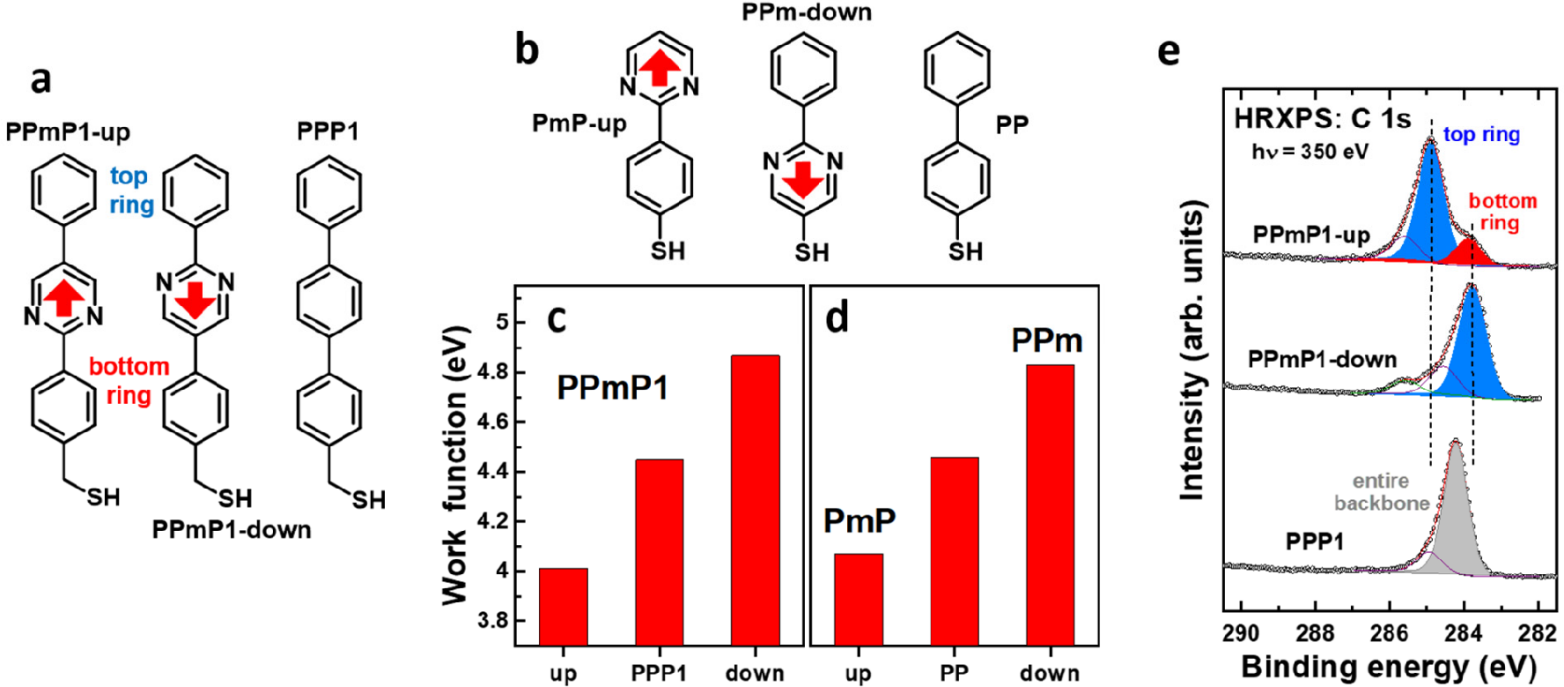
(a and b) Three- and two-ring SAM-forming molecules with embedded dipolar pyrimidine groups along with their names used for the remainder of the manuscript (P = phenyl, Pm = pyrimidine, 1 = single methylene spacer, up/down = direction of dipole moment (red arrows) relative to the anchoring group). (c and d) WFs of the SAMs grown on Au(111) substrates for the three-ring (c) and two-ring (d) systems. (e) X-ray photoelectron (XP) spectra of the PPP1-based SAMs; the peaks associated with the top and bottom rings are marked in blue and red. Adapted with permission from refs (1 and 4). Copyright 2015 Wiley-VCH (ref (1)). Copyright 2018 The Authors (ref (4)). Published by Wiley under a Creative Commons Attribution 4.0 International (CC BY 4.0) License. https://creativecommons.org/licenses/by/4.0/.

As an alternative approach, we designed a functional aromatic SAM system with an embedded dipole by substituting the central ring of 4,4′-terphenylmethanethiol (PPP1)^25^ with a dipolar pyrimidine moiety (Figure 1a).^1^ The SAMs fabricated using the molecules from Figure 1a were characterized in detail by a variety of complementary experimental techniques, verifying that their packing density and molecular organization are hardly affected by the presence of the pyrimidine groups. As expected, the WFs of the SAMs varied systematically, with changes relative to the nonpolar PPP1 SAM amounting to +0.43 eV (for the PPmP1-down SAM) and −0.55 eV (for the PPmP1-up SAM), consistent with the orientation of the pyrimidine dipoles (see Figure 1c). Another “electrostatic effect”, reported initially in ref (18), is a shift between the C 1s core-level energies of chemically identical moieties belonging to the regions above and below the dipole layers, as shown for the example of the PPP1-derived SAMs in Figure 1e. Again, the direction of the shift correlates with the direction of the dipole moments.

As a variant of the PPP1-based SAMs, analogous two-ring systems were designed (see Figure 1b).^4,26^ They feature noticeably shorter and fully aromatic molecular backbones, while preserving the electrostatic properties of the PPP1-type systems (see Figure 1d and ref (26)). To broaden the range of substrates onto which such SAMs can be grown, we also synthesized and successfully tested analogous molecules with phosphonic acid anchoring groups suitable for oxide substrates.^27^

**Figure 2:**
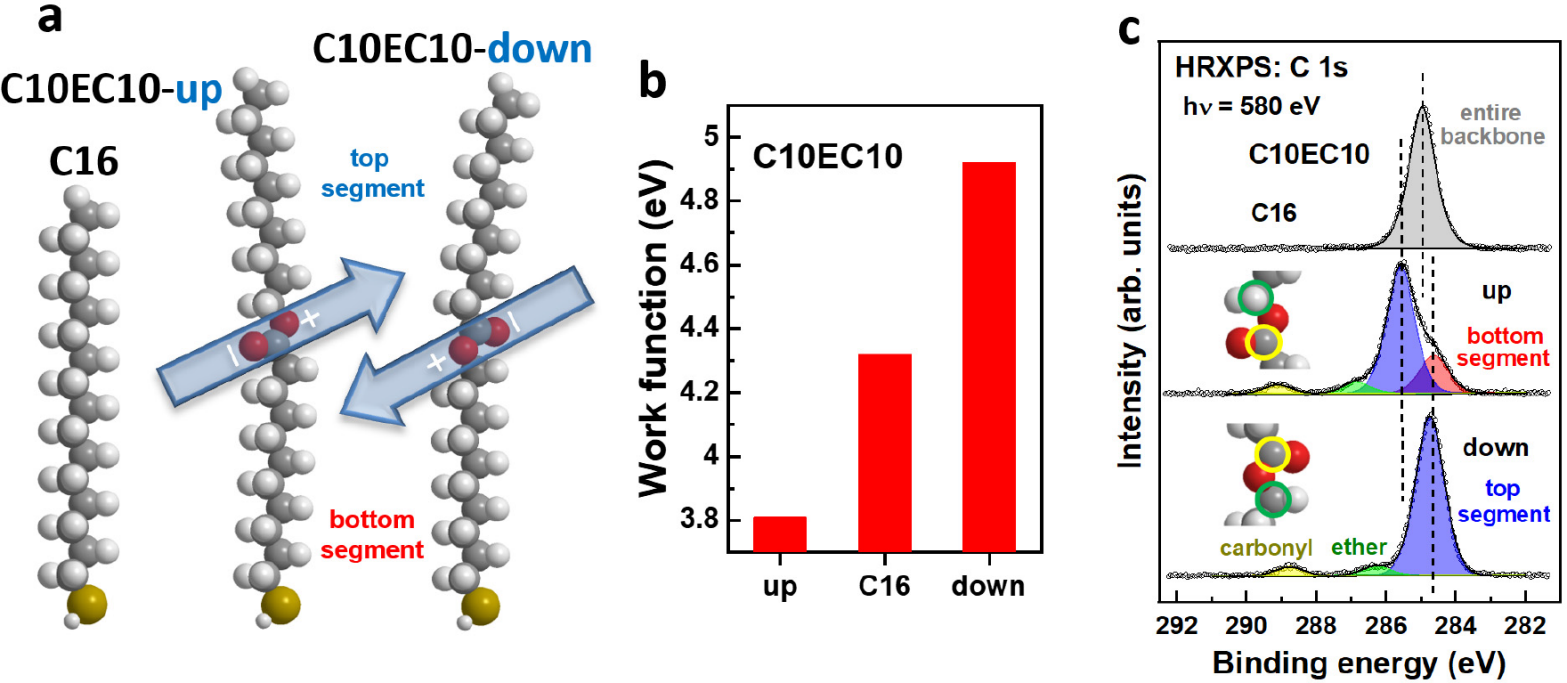
(a and b) Representative mid-ester substituted molecules and reference C16 system; the direction of the dipole moment relative to the molecular backbone is schematically shown. (b) SAM-induced WF changes and (c) C 1s XP spectra of the three systems shown in panel a. Individual peaks in the spectra are color-coded, accompanied also by the schematic drawings of the embedded groups. Adapted with permission from ref (28). Copyright 2017 American Chemical Society.

In view of the results for the pyrimidine-containing systems, we revisited the mid-ester substituted alkanethiolate SAMs mentioned above.^28^ Due to the inferior electrical conductance of aliphatic compared to aromatic chains, they are of less importance for electronic applications. Still they are valuable for basic research. Thus, we tested a variety of molecules with an ester group embedded between bottom and top alkyl segments of varying lengths, viz. HS(CH_2_)_*n*_COO(CH_2_)_*m*−1_CH_3_ (CnECm). In these systems, the situation was complicated by the fact that the dipole moments of the embedded esters are strongly inclined relative to the molecular axes (see Figure 2a). Considering the significant tilt of the molecules within the SAMs,^18,28^ this results in a delicate dependence of the electronic structure of the monolayers on the details of the film structure (see ref (28)). Nevertheless, the WF shift by the SAMs could in most cases be correlated with the orientation of the ester groups within the backbone, as demonstrated in Figure 2b for the representative C10EC10 monolayers: the WF shift with respect to the nonpolar hexadecanethiolate (C16) film was −0.5 eV for the “up” and +0.6 eV for the “down” orientations of the ester groups (Figure 2b). Again, an analogous behavior was observed for the C 1s XP spectra as shown in Figure 2c.

## Rationalizing the Changes in the SAMs’ Electronic Properties Originating from the Embedded Dipoles

**Figure 3:**
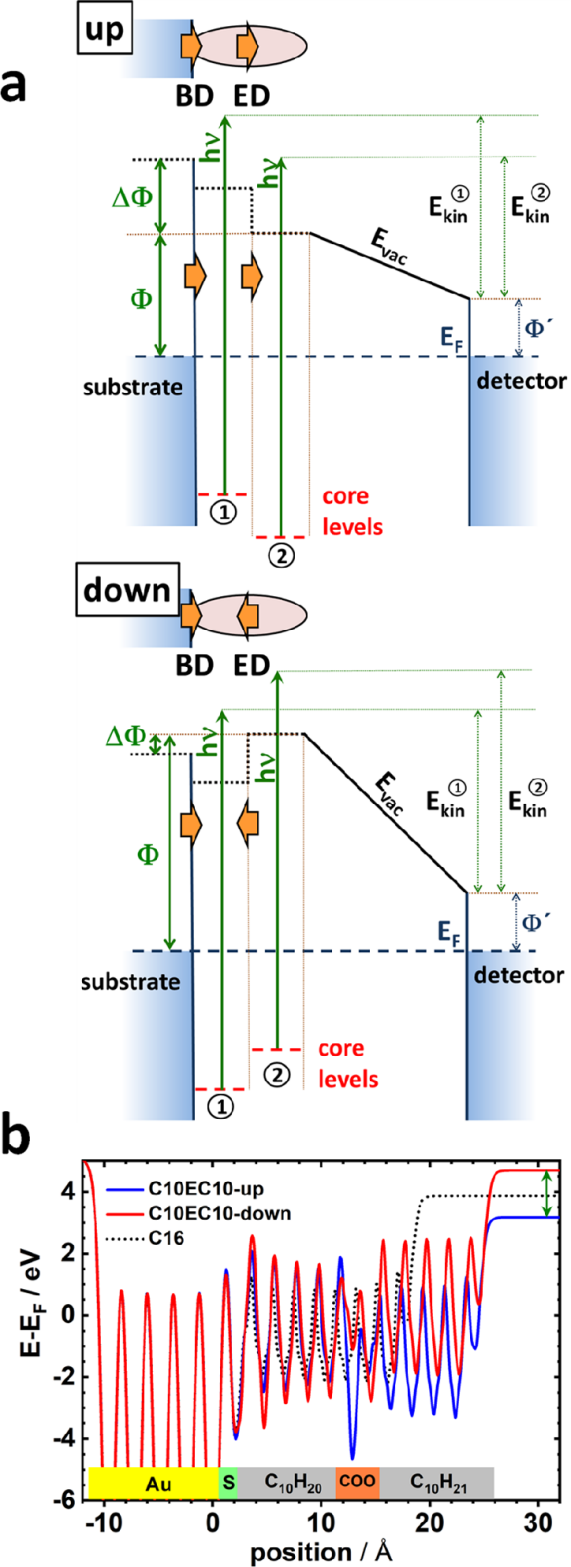
(a) Schematics of the electronic structure of the interface between a metal substrate and an embedded-dipole SAM comprising two dipole layers due to the bond dipoles (BDs) and the embedded dipoles (EDs). The SAM-induced change in the WF, ΔΦ (determined by the change in the offset between the Fermi level (*E*_F_) and the vacuum level (*E*_vac_) right above the sample), and the change in the core-level energies and the resulting kinetic energies of the photoelectrons (*E*_kin_^1^ and *E*_kin_^2^) are illustrated for the up and down orientations of the embedded dipoles. (b) DFT-calculated electrostatic energy averaged over a plane parallel to the interface for alkanethiolates containing an embedded ester group in two different orientations and for a C16 SAM as reference. Adapted with permission from refs (2 and 28). Copyright 2016 and 2017 American Chemical Society.

To understand the origin of the dipole-induced shifts of the WF and the XP spectra, one has to consider how extended arrays of dipoles impact the electrostatic potential: While an isolated dipole changes the electrostatic energy only in its immediate vicinity, the superpositions of the electric fields of an extended sheet of dipoles result in a step in the electrostatic energy such that the energies of the electronic states on the two sides of the dipole sheet are shifted relative to each other.^29^ This is illustrated schematically in Figure 3a. The energy shift due to these collective (or cooperative) electrostatic effects^30−32^ is proportional to the dipole density, and the spatial extent of the potential step is significantly smaller than the interdipole distance. For an embedded-dipole SAM on a surface, the first step in energy is caused by the bond dipole (BD in Figure 3) due to the polar anchoring groups and the bonding-induced charge rearrangements. It shifts the internal energy reference of the material (dotted black line in Figure 3a) and the core levels of the atoms within the SAM (dashed red lines) relative to the respective quantities of the substrate. A second step is then induced by the layer of the embedded dipoles (EDs in Figure 3), which causes another change of the aforementioned energies. As net effect of both dipole layers, one observes a change in the WF (ΔΦ). The embedded-dipole layer induces also a relative shift of the measured positions of the XP peaks of chemically identical atoms on the two sides of the layer (cf., situation depicted in the two panels of Figure 3a).

These dipole-induced energy shifts are illustrated also in Figure 3b, which shows the plane-averaged electrostatic energy (relative to the Fermi level) for the C16 SAM and for the C10EC10 SAMs with different dipole orientations:^28^ In the region between the substrate and the ester layer, the (average) electrostatic energies of all three systems are very similar (deviations within the SAMs arise from somewhat different tilts of the molecular backbones). Conversely, in the region between the ester layer and the SAM–ambient interface, the plane-averaged electrostatic energies differ significantly for the two dipole orientations. The difference prevails above the SAM, where the electrostatic energy corresponds to the vacuum energy and the energetic shifts correlate with the different WF changes.

**Figure 4:**
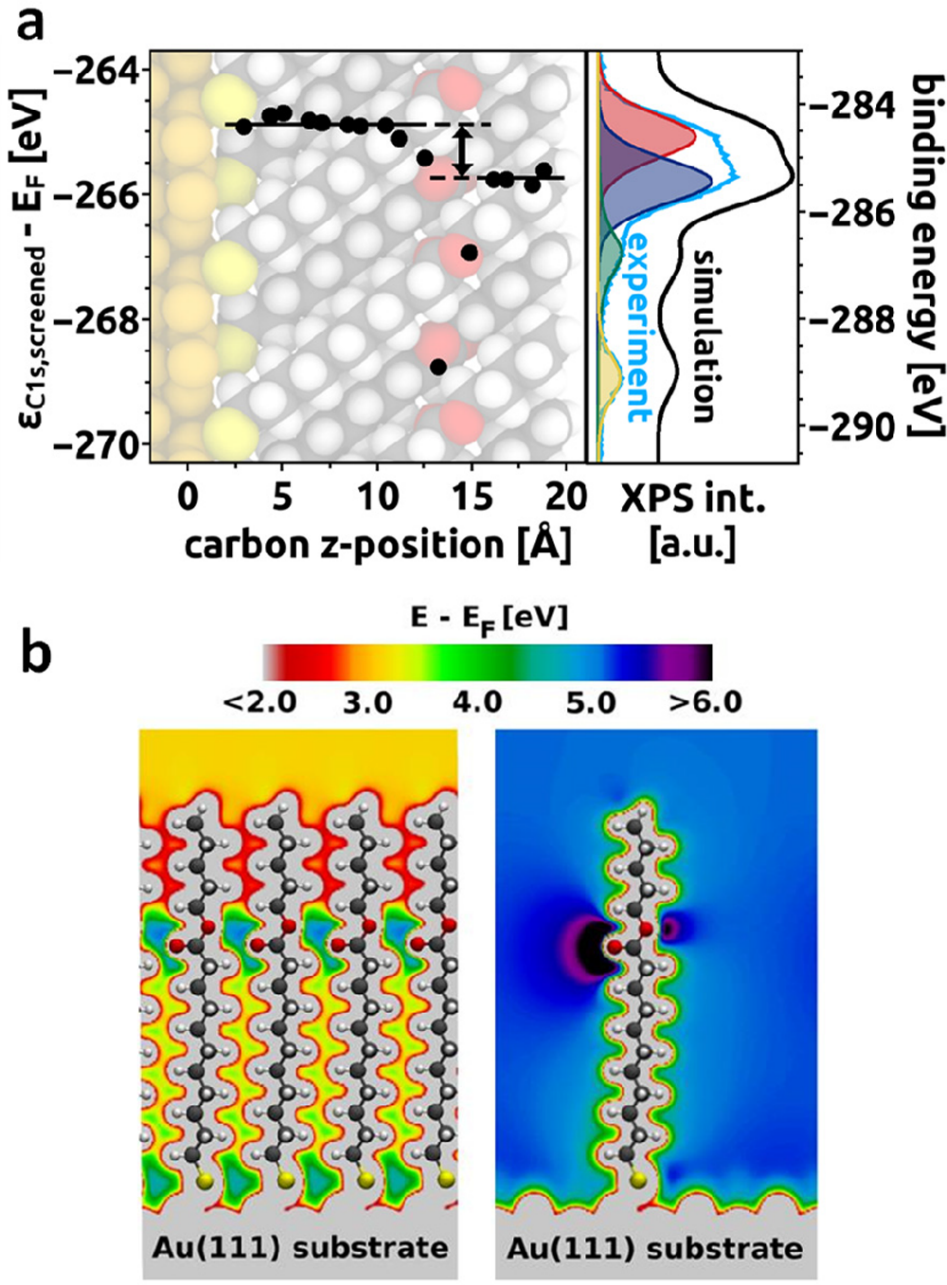
(a) DFT-calculated C 1s core-level energies in an alkanethiolate SAM containing an embedded ester group (C10EC5-up, left panel) with the resulting simulated XP spectrum compared to the actual experimental data (right panel). For further details see ref (2). (b) Comparison of the calculated electrostatic energy for a densely packed C10EC5-up SAM and for an isolated, upright-standing C10EC5-up molecule on a Au substrate. Adapted with permission from ref (2). Copyright 2016 American Chemical Society.

The left panel in Figure 4a illustrates the calculated shifts of the core-level energies due to the embedded dipoles in a mid-ester substituted alkanethiolate SAM. It confirms the above description of the overall situation, while the right panel illustrates that the calculated shifts very well reproduce the experimental observations (for a more in-depth discussion see ref (2)). Finally, Figure 4b illustrates the collective nature of the observed shifts, as a far-reaching (global) shift in the electrostatic energy (cf., color code) is induced only for a densely packed dipole layer, while an isolated ester group merely modifies the electrostatic energy in its immediate vicinity.

## Mixed Embedded-Dipole SAMs and the Continuous Tuning of the Work Function

**Figure 5:**
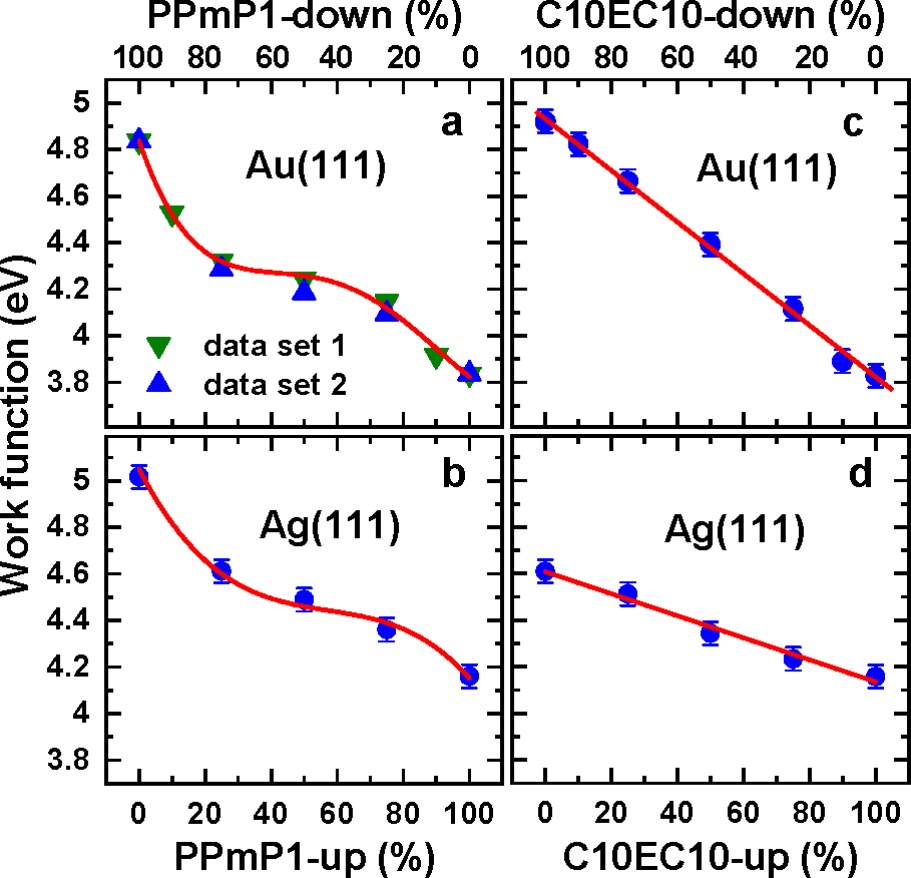
Dependence of the WF of the single-component and mixed PPmP1-up/down (a and b) and C10EC10-up/down (c and d) SAMs on Au(111) (a and c) and Ag(111) (b and d) on the concentration of PPmP1-up (a and b) or C10EC10-up (c and d) molecules in the solutions from which the monolayers were grown. The red lines serve as guides to the eye highlighting the character of the dependencies. Adapted with permission from refs (3), (36), and (37). Copyright 2016, 2017, and 2018 American Chemical Society.

Generally, not only fixed but also tunable WF values can be achieved by combining molecules with differently oriented dipole moments. This is usually realized in mixed monolayers consisting of molecules with different polar tail groups.^20,33−35^ Similarly, when growing mixed monolayers of embedded-dipole SAMs consisting of “up” and “down” molecules, it is possible to continuously tune the WF of the system between the values of the homogeneous SAMs, as illustrated in Figure 5.^3,36,37^ Such mixed monolayers were prepared by coadsorption, mixing the respective molecules in solution. Here, we observed distinctly different behaviors for the pyrimidine- and ester-based embedded-dipole SAMs. For the PPmP1-up/down system, the composition of the mixed SAMs differs from the composition of the solutions with a preference for 50–50% mixing ratios, as is apparent from the sigmoid WF-vs-composition curves in Figure 5a and b. We attribute this to the dipole–dipole interaction between the pyrimidine rings, which constitute a significant part of the molecular backbones.^3^ In contrast, for the C10EC10-up/down case, the composition of the mixed SAMs mimics that of the parent solution, resulting in linear WF-vs-composition curves (Figure 5c and d).^37^ This is attributed to the strongly inclined ester dipoles (vide supra) and to the ester groups constituting only a small part of the molecular chains. Another important aspect is a noticeable reduction of the accessible WF range for C10EC10/Ag compared to C10EC10/Au (Figure 5c and d). This is a consequence of the reduction of the molecular tilt from ∼30° to ∼12° when replacing Au(111) by Ag(111), which causes a reduced dipole component perpendicular to the substrate surface (for more details see ref (37)). In passing, we note that the WF tuning has also been tested for PPm-down and PmP-up SAMs, with similar results as for the PPmP1-up/down system.^38^

**Figure 6:**
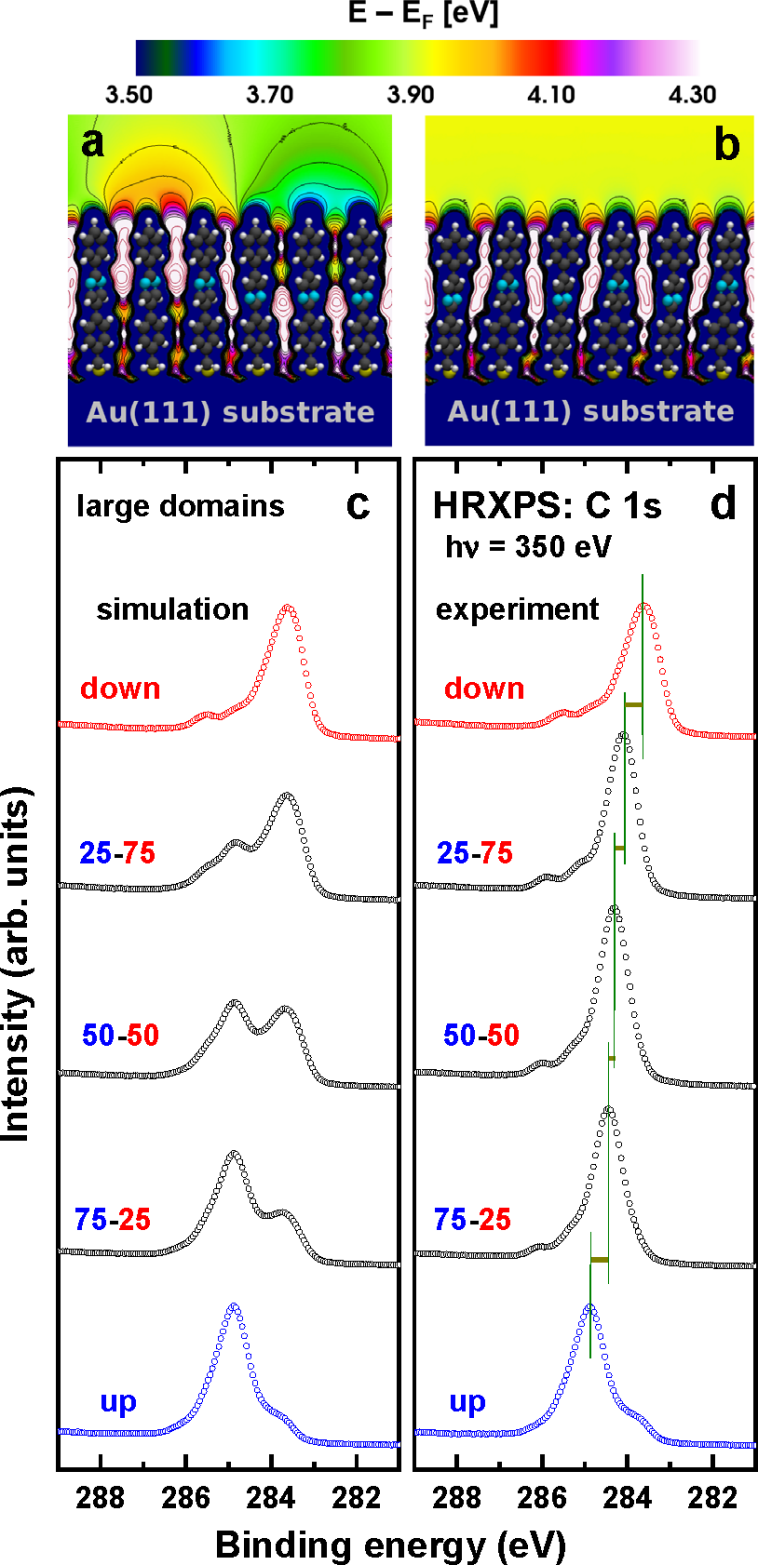
Electrostatic energy plotted for the models of phase separated (a) and homogeneous (b) SAMs comprising a 50:50 mixture of PPmP1-up and PPmP1-down molecules. (c) Weighted superposition of the C 1s XP spectra of the single-component PPmP1-up and -down SAMs, mimicking large scale phase separation in the mixed films. (d) Experimental C 1s XP spectra of the mixed PPmP1-up/down SAMs. The dominant peak corresponds to the core-level excitations for C atoms in the top ring; its position is shifted electrostatically by the embedded dipoles. Weights and compositions in parts c and d are color-coded. Adapted with permission from ref (3). Copyright 2016 American Chemical Society.

When tuning the WFs of electrodes, a homogeneous mixing of the two components is highly beneficial to avoid injection hot spots due to a possible phase separation. In the present systems, XPS allows an identification of a possible phase separation, as the core-level binding energies are much more sensitive to local variations of the electrostatic energy than the WFs.^3^ These local variations are compared in Figure 6 for a model system built from three molecule wide stripes consisting of identical molecules (Figure 6a) and for a homogeneously mixed SAM (Figure 6b). While the situation in Figure 6a results in significantly broadened XP spectra or even a double-peak structure (depending on the stripe width), for the case depicted in Figure 6b a peak shift without any broadening is calculated.^3^ The formation of even larger domains can be modeled by weighted superpositions of the spectra of the single-component SAMs, as illustrated in Figure 6c. Notably, as shown in Figure 6d, in the actual experiments the C 1s peak does not broaden at all and varies continuously with the PPmP1-up/down ratio, mimicking the trend of the WF change (including its sigmoid character). This is a clear indication for a homogeneous mixture of the “up” and “down” molecules.^3^

## Charge Transport through Embedded-Dipole SAMs

**Figure 7:**
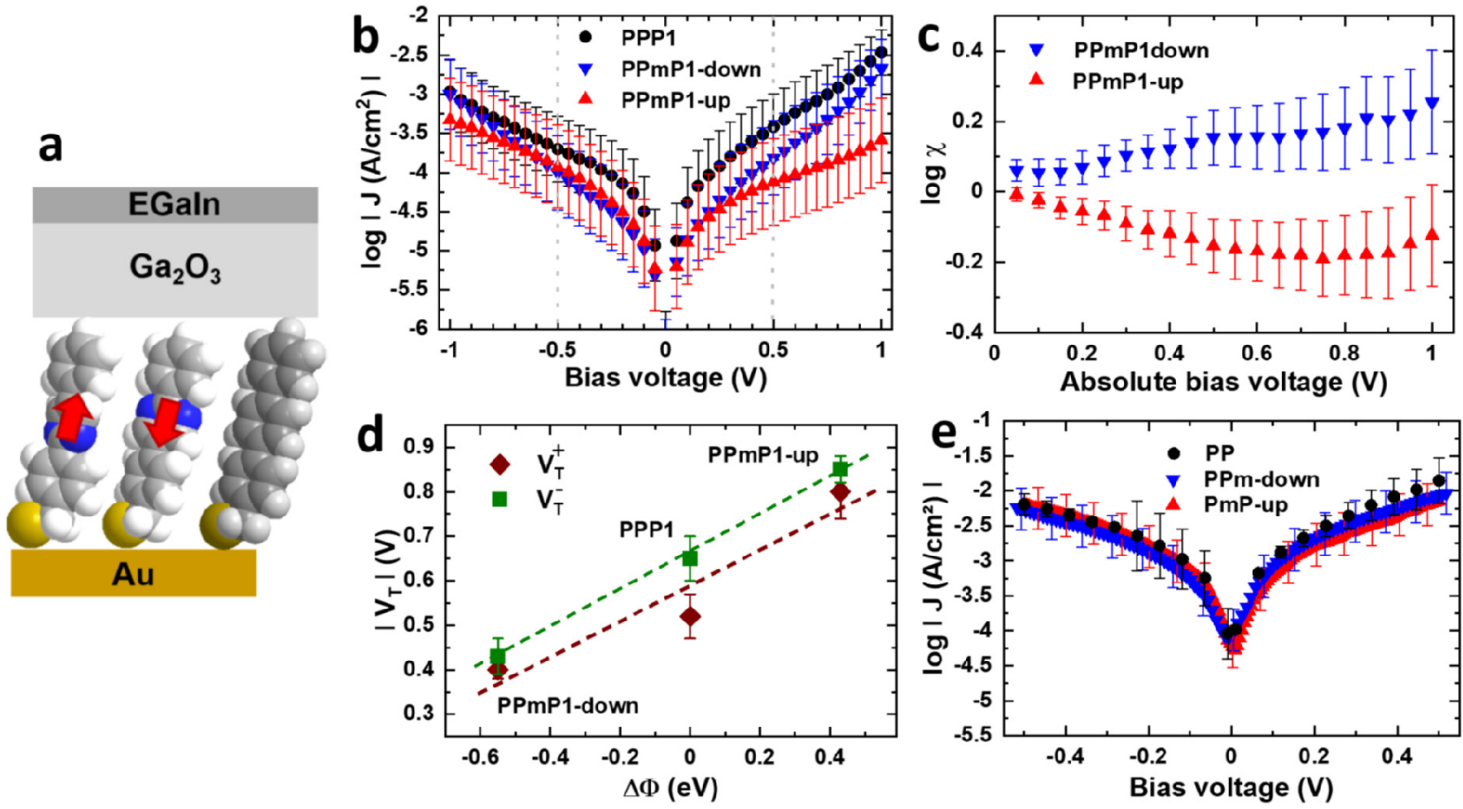
(a) Schematic structure of the Au/SAM//Ga_2_O_3_/EGaIn junctions featuring the PPP1-based SAMs; EGaIn is a eutectic GaIn alloy covered by a thin (∼0.7 nm) oxide layer. (b) *I*–*V* curves and (c) current asymmetry (log of the ratio of the currents for forward and reverse bias as a function of the absolute value of the bias) for junctions comprising PPmP1-up and PPmP1-down SAMs. Panel d illustrates transition voltage (*V*_T_) vs WF change for these junctions. *V*_T_ was measured for both positive (*V*_T_^+^) and negative (*V*_T_^–^) bias. (e) *I*–*V* curves for junctions featuring the PP-based SAMs consisting of molecules with only two aromatic rings and no methylene spacer (see Figure 1b). Adapted with permission from refs (39), (40), and (26). Copyright 2016 Authors (refs (39) and (40)). Published by Royal Society of Chemistry under a Creative Commons Attribution 3.0 International (CC BY 3.0) License. https://creativecommons.org/licenses/by/3.0/. 2018 American Chemical Society (ref (26)).

An important property of SAMs is their ability to conduct charges. Thus, the conductivity of dipolar SAMs and its variation by electrostatic effects is of distinct importance, especially as it can directly affect the performance of devices containing such SAMs. The *I*–*V* curves of the PPP1-based monolayers recorded in a two-terminal junction setup (Figure 7a) show a minor effect of the embedded dipole on the magnitude of the current density (Figure 7b) and, consequently, on the conductivity of the SAMs.^39^ There is, however, a certain asymmetry in the conductivity (Figure 7c) ascribed to the collective action of embedded dipoles directed either parallel or antiparallel to the transport direction.^40^ The mechanism behind this action is the bias-induced (de)localization of the frontier electronic states that has a direct impact on charge transport.^40^

The *I*–*V* curves also allow extraction of important parameters such as the transition voltage (*V*_T_), which is regarded as an approximate measure for the tunneling barrier height arising from the energy offset between the frontier orbitals of a SAM and the Fermi level of the electrode.^41,42^ Embedded-dipole SAMs are particularly interesting for determining the dependence of *V*_T_ on the intrinsic characteristics of the molecular monolayer, as differences in the dipole should not impact the properties of the interfaces with the two electrodes.^39^ Interestingly, when comparing the PPmP1-up, PPmP1-down, and PPP1 SAMs, one observes a linear correlation between *V*_T_ and the SAM-induced WF shift (see Figure 7d). A similar linear correlation is also observed between *V*_T_ and the calculated injection barrier between the valence band of the SAMs and the substrate Fermi level. The latter is impacted by the embedded dipoles via the partial localization of the frontier states either below or above the embedded dipoles combined with the modification of the potential landscape caused by the dipole layer (see ref (39)).

While PPP1-derived SAMs are best suited for the above experiments, their rather low electrical conductance can be problematic for their application in organic electronic devices. As these low conductances are primarily due to the rather long backbones and the nonconjugated methylene linker, the PP-derived systems from Figure 1b should significantly improve the situation. The data in Figure 7c show that these systems, indeed, feature massively increased current density at a given bias voltage.^4,26^ This makes these SAMs particularly attractive for applications in macroscopic electronic devices, as will be illustrated in the next section.

Prior to that it should be mentioned that the electronic transport properties of SAMs with embedded-dipole groups have also been studied for systems other than pyrimidine-containing oligophenylenes. For example, Yoon et al. reported hardly any effect for a variety of mid-substituted alkanethiolate SAMs,^21^ whereas a small but distinct effect (compared to the reference, nonpolar film) was observed for partially fluorinated alkanethiolates by Bruce et al.^22^ In the studies by Lee et al. on embedded-dipole SAMs containing pyrimidines, the impact of varying anchoring groups clearly dominated over the consequences of the embedded dipole.^43^ As an alternative approach, we employed the so-called core-hole clock method specifically adapted to SAMs^44,45^ to a series of fluorine-side-decorated benzonitrilethiolates and -selenolates with variable dipole moments. Also here we observed hardly any impact of the variation of the dipole direction and magnitude on the measured electron transfer times.^46^

## Modifying Electrode Properties with Embedded-Dipole SAMS

**Figure 8:**
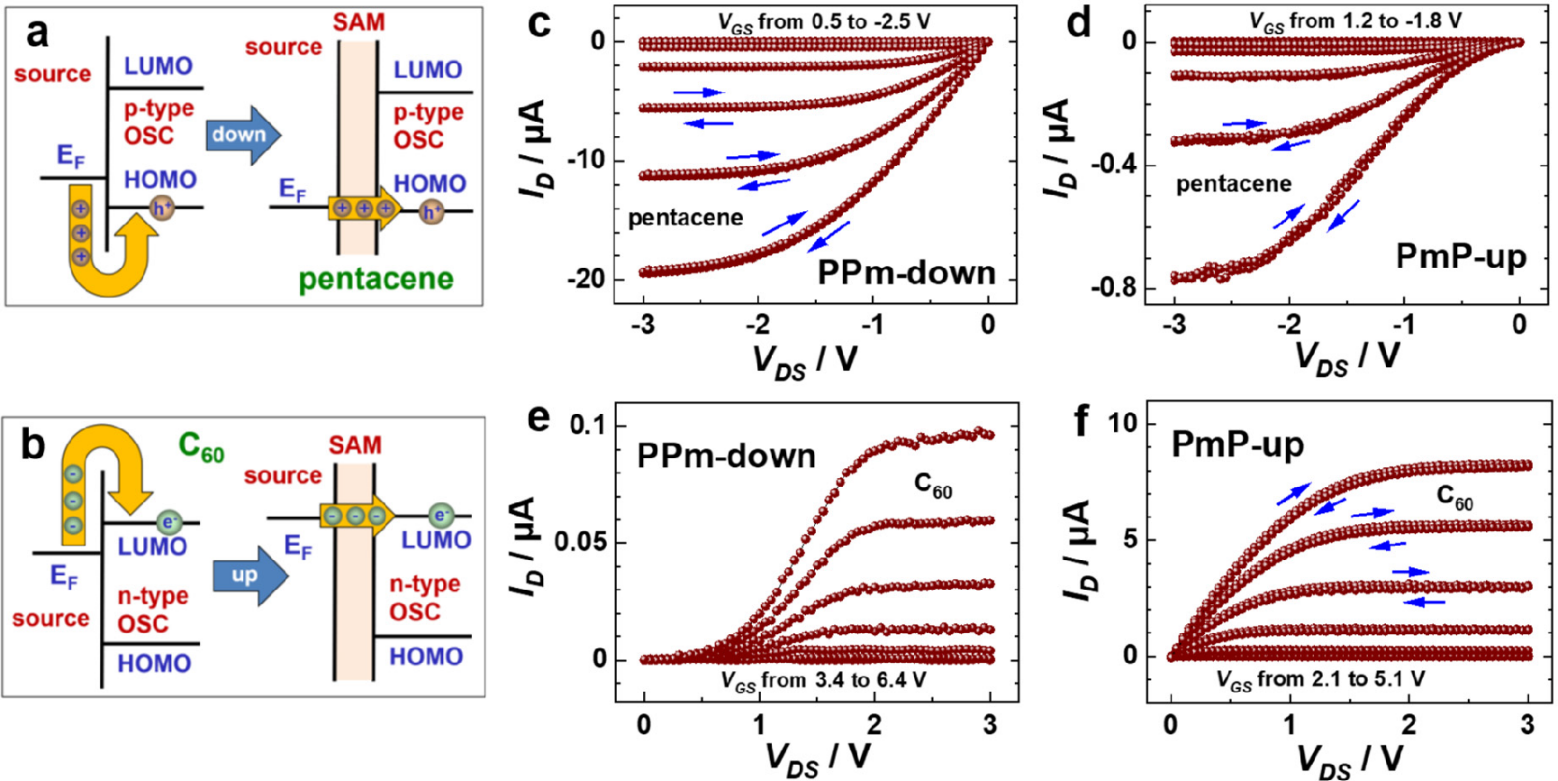
(a and b) Schematic of the energy level alignment between the Au electrode and p- and n-type organic semiconductor before and after introducing suitable up/down embedded-dipole SAMs. (c and d) Typical output characteristics of p-type pentacene OTFTs with the Au electrodes modified by the PPm-down (c) and PmP-up (d) SAMs. (e and f) analogous characteristics of n-type C_60_ OTFTs with the Au electrodes modified by the PPm-down (e) and PmP-up (f) SAMs. Adapted with permission from ref (4). Copyright 2018 The Authors. Published by Wiley under a Creative Commons Attribution 4.0 International (CC BY 4.0) License. https://creativecommons.org/licenses/by/4.0/

**Figure 9:**
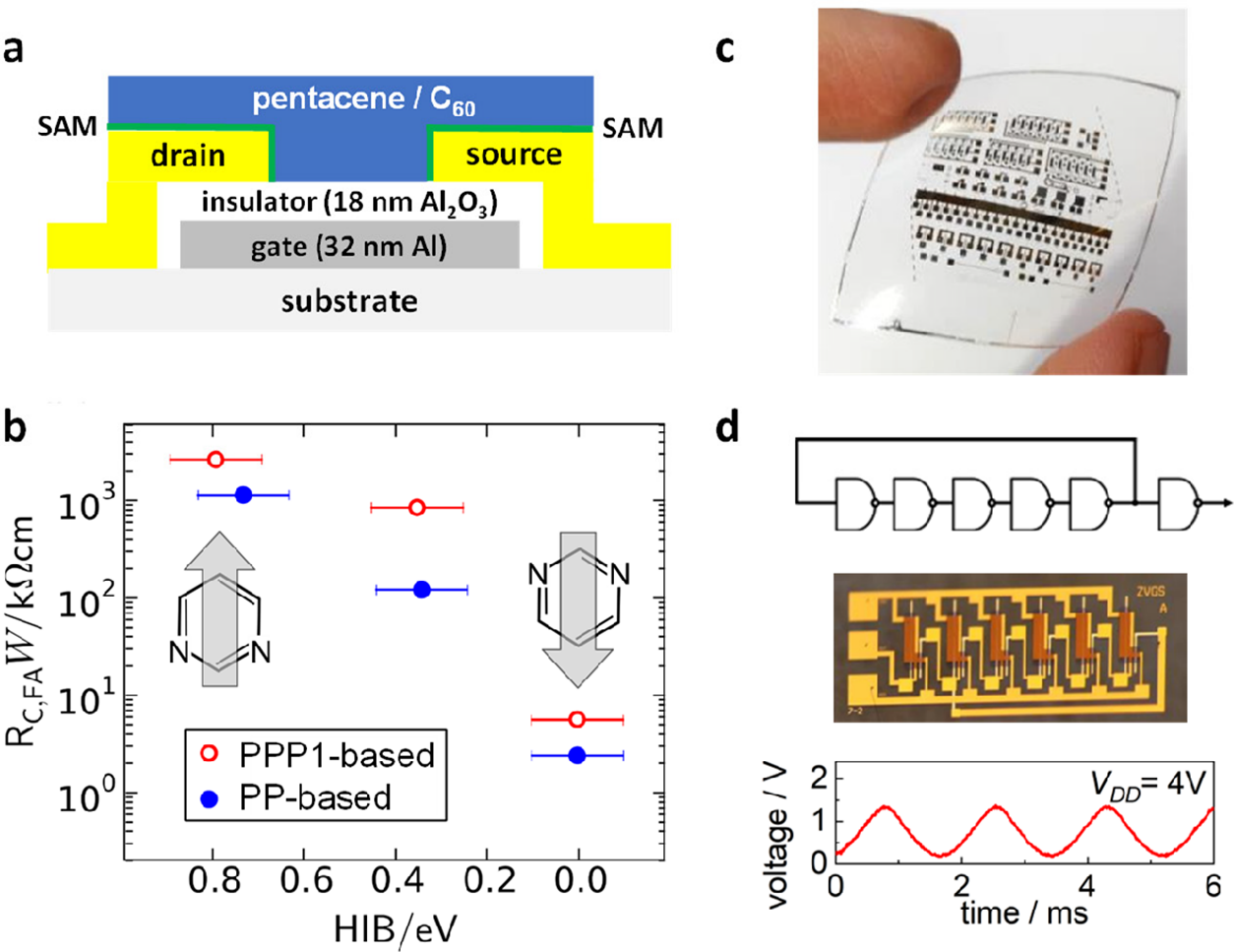
(a) Schematic structure of an OTFT with electrodes modified by distributed-dipole SAMs. (b) Contact resistances of the source electrodes in pentacene-based transistors as a function of the orientation of the embedded dipoles and the length of the backbones. (c) Devices with SAM-modified electrodes on a flexible substrate and (d) a 5-stage ring oscillator with a buffer stage fabricated on a flexible substrate. Adapted with permission from ref (4). Copyright 2018 The Authors. Published by Wiley under a Creative Commons Attribution 4.0 International (CC BY 4.0) License. https://creativecommons.org/licenses/by/4.0/.

The significant work-function changes induced by embedded-dipole SAMs shown in Figures 1c and d and 2b suggest that such systems should be very well suited for modifying the barriers for injection from metal contacts into active organic semiconductor layers. This is conceptually illustrated in Figure 8a and b, where it is shown that SAMs with a dipole-“down” orientation promote hole injection (Figure 8a), while dipole-“up” SAMs are beneficial for contacts injecting electrons. The considerations in the previous section imply that among the systems studied here, the PPm-down and PmP-up SAMs would be best suited for the purpose, as they display the highest conductances. Thus, we applied these SAMs (as well as the PPmP1-up and PPmP1-down systems) in both n- and p-type transistors to modify the source and drain electrodes, as illustrated in Figure 9a.^4,26^ As shown in Figure 8 c–f, this yielded exactly the expected results: In the p-type transistors (with pentacene as active material) the current increased massively for the “proper” dipole orientation despite the essentially equivalent growth of the pentacene layers on the embedded-dipole SAMs.^4^ A similar effect was observed for the n-type devices relying on C_60_, albeit for a flipped dipole orientation. In this context it is worthwhile to mention that all devices used Au as electrode material. This is insofar remarkable, as—to the best of our knowledge—Au (due to its high WF) has not been applied as electrode material in n-type OTFTs before.

A more in-depth analysis of the device characteristics of the pentacene-based transistors (Figure 9a) reveals that the contact resistances are more than two orders of magnitude smaller when using the dipole-“down” SAMs compared to employing the dipole-“up” systems; additionally, contact resistances are clearly smaller for PP-based than for PPP1-based SAMs (see Figure 9b).^4^ Notably, a similarly beneficial effect of the embedded-dipole SAMs is observed also on flexible substrates (see Figure 9c) and for more complex circuits, such as ring oscillators (see Figure 9d).^4^ Interestingly, besides this application in organic electronics, the SAMs discussed here also had a highly beneficial effect as electrode modifiers in transistors employing 2D semiconductors (in particular MoS_2_) as the active material.^47^

## Perspectives: Distributed Dipole SAMs and Polar Linkers in Complex Framework Materials

When realizing alternative embedded-dipole systems, one possibility is to vary the anchoring chemistry (using selenolates instead of thiolates or employing carboxylic acids for bonding to Ag(111) and oxides). Alternatively, the chemical nature of the polar entities can be varied. An option would be partly fluorinated rings, which were, for example, employed in the embedded-dipole framework materials discussed below.

**Figure 10:**
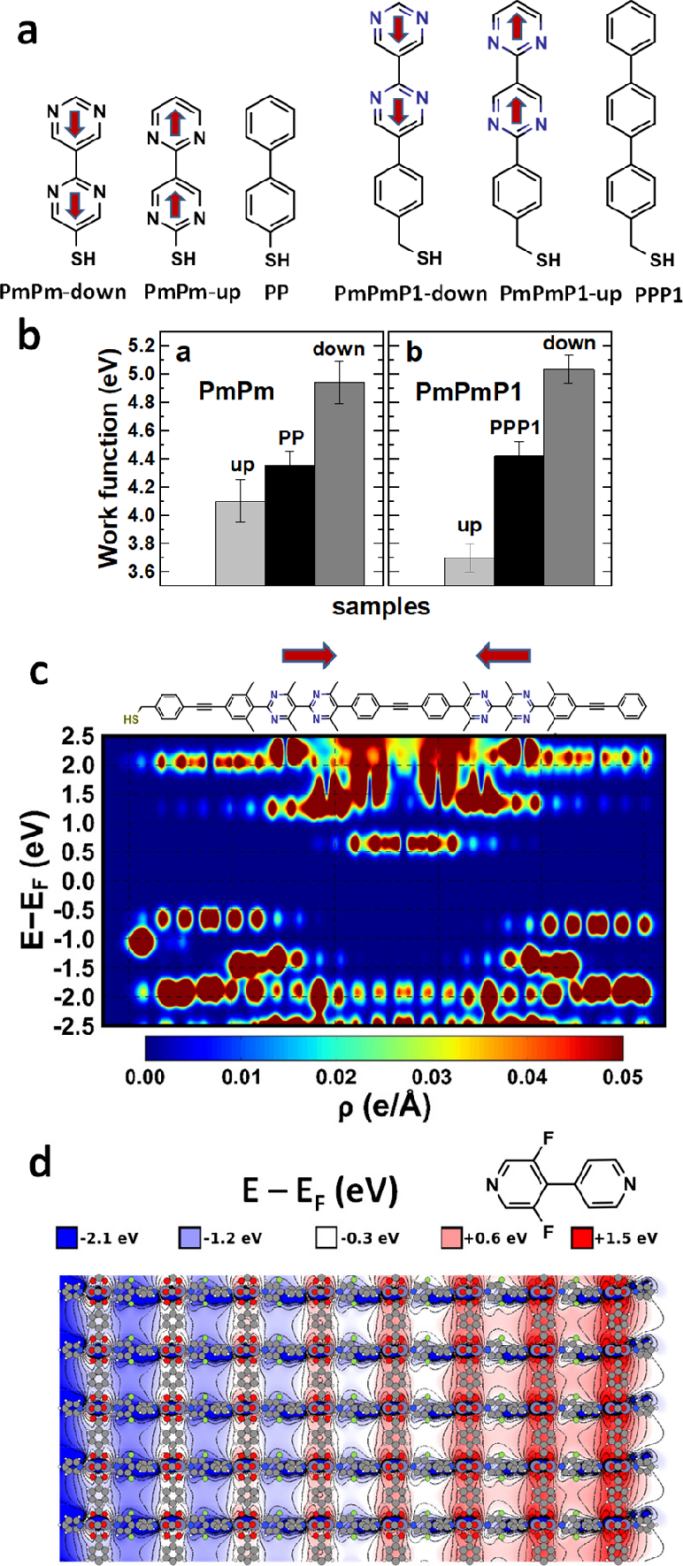
(a) Chemical structures of the distributed dipole SAMs studied in ref (48) and (b) WF modifications they induce when adsorbed onto a Au(111) surface. (c) Density of states (as a function of position and energy) of a monolayer consisting of more complex embedded-dipole molecules containing oppositely oriented polar groups consisting of bipyrimidine units. The alignment of the dipoles creates a quantum well for electrons in the central section of the monolayer. (d) Evolution of the DFT-calculated electrostatic energy for a MOF thin film consisting of 1,4-benzenedicarboxylate-linked Zn-paddlewheel sheets connected by seven layers of apical, polar (partly fluorinated) bipyridine linkers (see top-right inset). Adapted with permission from refs (48−50). Copyright 2020 American Chemical Society (ref (48)). Copyright 2015 (ref (49)) and 2020 (ref (50)) The Authors. Published by Wiley and MDPI under a Creative Commons Attribution 4.0 International (CC BY 4.0) License. https://creativecommons.org/licenses/by/4.0/.

A step beyond embedded-dipole SAMs involves including several consecutive polar entities into the molecular backbones to realize so-called distributed-dipole SAMs (structures of actually synthesized molecules are shown in Figure 10a). Initial experiments with such SAMs have already been performed.^48^ Unfortunately, the measured work-function changes induced by these SAMs were somewhat inconclusive: While for all SAMs the simulations predicted essentially a doubling of the work-function change compared to the systems with only one pyrimidine in the backbone, in the experiments this was observed only for the PmPmP1-up SAM (see Figure 10b), which could be caused by a variety of reasons, as discussed in detail in ref (48). This suggests that the distributed dipole concept is promising, but to lift its full potential more efforts will be necessary. Eventually, distributing several dipoles along molecular backbones in more complex patterns could even allow the realization of SAMs in which electrostatically designed quantum cascades or quantum wells exist (see Figure 10c).^49^

As a last aspect it should also be mentioned that the use of embedded-dipole linkers has also been suggested for porous metal–organic frameworks (MOFs)^50^ and covalent organic frameworks (COFs).^51^ There they could be used to induce potential gradients^50^ or to produce more complex quantum structures.^51^ As an example, Figure 10d shows the gradient of the electrostatic energy that is generated in a MOF in which the apical bipyridine linkers have a dipole moment due to partial fluorination.^50^ The key challenge for the experimental realization of such systems is that one must realize an asymmetric bonding of the linkers in order to align their dipoles (see discussion in ref (50) for details). The first successful steps in this direction have recently been made for 1,4-biphenyldicarboxylate-linked Cu-paddlewheel sheets connected by polar apical linkers,^52^ but in these experiments the realized degree of dipole alignment was still rather low.

## Concluding Remarks

The above results show that embedding polar entities into the backbones of SAM-forming molecules is a versatile tool for realizing SAMs that enable tuning the electronic properties of interfaces. The major advantage of this approach is that it allows disentangling the electrostatic engineering of interfaces from chemical modifications of the SAM surfaces. This is particular promising for optimizing electrodes in devices used in organic electronics and photovoltaics, as it allows tuning injection barriers without adversely impacting the growth of active layers.

Embedded-dipole SAMs are also interesting in the context of basic research as they further our understanding of how the electronic properties of an interface depend on the presence of ordered polar layers; they provide insights into how these polar layers and the associated orbital localization impact parameters relevant for ballistic charge transport through monolayers (such as transition voltages and rectification ratios); they also reveal that core-level binding energies are affected not only by chemical shifts but also to a comparable extent by dipole-induced modifications of the electrostatic potential. The latter allows a rational interpretation of XP data for polar systems and, under certain circumstances, enables a study of the homogeneity of mixed polar films at the individual molecule level by XPS.

A logical extension of embedding individual polar units into molecules is distributing several polar entities along molecular backbones. The first results on such distributed dipole SAMs are promising, but further efforts are necessary for realizing the full potential of this approach. Notably, both embedded and distributed dipole architectures are not limited to SAMs but can also be utilized in more complex and more extended systems, such as electrostatically engineered, porous framework materials.
